# Psychological pain tolerance and its associated factors among hospitalized patients with enterstomy in China: a cross-sectional quantitative study

**DOI:** 10.3389/fpsyg.2026.1694379

**Published:** 2026-03-31

**Authors:** Jie Song, Jun Qu, Liping Li, Peng Sun, Guiqiu Fang, Yufeng Cui, Xiaochun Tang, Jun Yin, Huilong Shen, Yuanzhi Li, Xiaoping Liu, Zhenfa Li, Weidong Gong, Zhangyi Wang

**Affiliations:** 1 Department of Nephrology, Tianjin Fourth Central Hospital, Tianjin, China; 2 Gastrointestinal Surgery/National Key Clinical Construction Specialist General Surgery, Affiliated Hengyang Hospital of Hunan Normal University & Hengyang Central Hospital, Hengyang, Hunan, China; 3 Dean's Office, Tianjin First Central Hospital, Tianjin, China

**Keywords:** associated factors, cross-sectional study, enterstomy, hospitalized patients, psychological pain tolerance, psychosocial adaptation, quality of life, self-care

## Abstract

**Background:**

The importance of psychological pain tolerance has been widely acknowledged in global studies, demonstrating its superior predictive validity for suicidal behaviors and quality of life outcomes. As a robust protective factor, enhanced psychological pain tolerance not only mitigates suicide risk but also improves adaptive coping strategies. However, existing research indicates that psychological pain tolerance among hospitalized patients with enterstomy remains underdeveloped. Furthermore, limited studies have examined the psychological pain tolerance and its associated influencing factors among hospitalized patients with enterstomy.

**Objective:**

To investigate psychological pain tolerance and its associated factors among hospitalized patients with enterstomy in China, thereby establishing an evidence-based foundation for developing individualized nursing interventions to manage psychological distress in enterostomy care.

**Methods:**

A cross-sectional design was employed, adhering to the quality reporting conformed to the STROBE Checklist. From March 2024 to May 2025, a total of 506 hospitalized patients with enterstomy were recruited from three tertiary grade-A hospitals in China. The Demographic Characteristics Questionnaire, Tolerance for Mental Pain Scale (TMPS), Ostomy Adjustment Inventory (OAI), Stoma Quality of Life (S-QOL), and Stoma Self-care Scale (SSCS) were used. Data were analyzed using descriptive statistics, univariate, correlation analyses, and multiple linear regression analysis.

**Results:**

The total score of psychological pain tolerance was 32.18 ± 10.42, psychosocial adaptation was 44.77 ± 18.56, quality of life was 49.18 ± 17.90, and self-care was 33.58 ± 11.42. Psychological pain tolerance was positively correlated with the scores for psychological adaptation (*r* = 0.674, *p* < 0.01), quality of life (*r* = 0.542, *p* < 0.01), and self-care (*r* = 0.513, *p* < 0.01), with positive correlations observed across all dimensions (*r* = 0.346–0.644, *p* < 0.01). Multiple linear regression analysis showed that age, gender, living status, time after enterstomy, psychological adaptation, quality of life, and self-care were the main factors associated with psychological pain tolerance among hospitalized patients with enterstomy, explaining 45.8% of the total variation (*p* < 0.05).

**Conclusion:**

The psychological pain tolerance among hospitalized patients with enterstomy was moderate, which need to be further improved. Age, gender, living status, time after enterstomy, psychological adaptation, quality of life, and self-care were the main associated factors of psychological pain tolerance among hospitalized patients with enterstomy. It is suggested that nursing staff should formulate personalized intervention programs according to the associated factors of psychological pain tolerance among hospitalized patients with enterstomy, so as to improve the quality of life among hospitalized patients with enterstomy.

## 1 Introduction

According to the Global Cancer Statistics 2020 released by the International Agency for Research on Cancer (IARC), colorectal cancer accounted for approximately 1.9 million new cases worldwide, representing the third most prevalent malignancy globally. Concurrently, mortality data revealed approximately 935,000 colorectal cancer-related deaths, positioning it as the second leading cause of cancer-related mortality (Sung et al., 2021). To address the dual challenges of elevated morbidity and mortality, surgical interventions involving temporary or permanent enterstomy formation remain the primary therapeutic approach for life preservation in colorectal cancer management (Zheng and Wang, 2021).

Recent epidemiological data indicate an annual increase of approximately 100,000 new enterstomy cases worldwide, with a notable demographic shift toward younger patient populations (Li et al., 2024). Postoperative adaptation challenges, including alterations in defecation patterns, physiological structure, and body image, coupled with postoperative disease-related distress and enterstomy management challenges, frequently result in compounded psychological burden. A cross-sectional study revealed that 56.8% of Chinese enterstomy patients exhibit clinically significant psychological distress (Chen et al., 2024), markedly exceeding the 24.2% prevalence observed in the general cancer population (Zhang, Y. N. et al., 2010).

Psychological pain, operationally defined as a multidimensional unpleasant emotional state characterized by anxiety, depressive symptoms, and existential concerns (Hamer et al., 2009; Meerwijk and Weiss, 2011), has been identified as both a precursor to maladaptive coping mechanisms and a critical predictor of suicidality (Lutzman and Sommerfeld, 2024). Emerging evidence suggests that assessing psychological pain tolerance—the capacity for emotional regulation and cognitive reappraisal of distressing experiences (Meerwijk et al., 2019)—provides superior predictive validity for suicidal behaviors and quality of life outcomes compared to traditional distress measurements (Becker et al., 2019). As a robust protective factor, enhanced psychological pain tolerance not only mitigates suicide risk but also improves adaptive coping strategies (Landi et al., 2023), while serving as a potential prognostic indicator for psychotherapeutic outcomes.

Current research on psychological pain tolerance predominantly focuses on populations with HIV/AIDS, depressive disorders, and oncological conditions, with a paucity of investigations addressing enterstomy patients. This study therefore aims to investigate psychological pain tolerance and its associated factors among hospitalized patients with enterstomy, thereby providing evidence-based insights for developing targeted nursing interventions and psychological support protocols.

## 2 Objective

To investigate the psychological pain tolerance and its associated factors among hospitalized patients with enterstomy, thereby establishing an evidence-based foundation for developing individualized nursing strategies that address psychological distress management in enterstomy care.

## 3 Methods

### 3.1 Study design and setting

This study used a descriptive cross-sectional design and was conducted in China. The quality reporting of the study adhered to the Strengthening the Reporting of Observational Studies in Epidemiology (STROBE) guidelines, which outline the necessary information to include in reports of cross-sectional studies.

### 3.2 Participants and sample

A non-probability convenience sampling method was employed to enroll eligible hospitalized patients with enterstomy from three tertiary grade-A hospitals in China between March 2024 and May 2025. The study established rigorous inclusion and exclusion criteria to ensure participant suitability. Inclusion criteria comprised: (1) Individuals who had undergone surgical creation of an enterostomy through the abdominal wall; (2) Patients with a minimum enterstomy duration of one month without reversal surgery; (3) Capability of providing informed consent and voluntarily agreeing to participate in all study procedures; (4) Absence of diagnosed intellectual disabilities combined with clinically stable condition permitting study participation. Exclusion criteria encompassed: (1) Subjects with documented psychiatric disorders or clinically significant cognitive impairments; (2) Patients presenting with severe comorbidities that could compromise study cooperation; (3) Concurrent participation in other clinical trials within the past one month.

G*Power 3.1.9.7 software (Faul et al., 2009) was used to calculate the minimum sample size required for *F*-tests [*α* error probability was 0.05, and power (1-*β* error probability) was 95%]. Because all variables involved of this study are continuous, therefore, the *F-*tests method was selected in G*Power 3.1.9.7 software, “Linear multiple regression: Fixed model, *R*^2^ deviation from zero” of statistical test was selected, “*A priori*: Compute required sample size - given *α*, power, and effect size” of type of power analysis was selected, and then the parameter effect size *f*^2^ = 0.15, *α* = 0.05, 1-*β* = 0.95 were set, and finally clicked “Calculate” to calculate the total sample size = 107, the minimum sample size of this study.

Considering the hospital and clinical realistic environment, 506 participants were included in this study in the end. And “*Post hoc*: Compute achieved power-given *α*, sample size, and effect size” of type of power analysis was selected. And then the parameter effect size *f*^2^ = 0.15, *α* = 0.05, total sample size = 506 were set, and finally clicked “Calculate” to calculate the 1-*β* = 1.00, which was greater than the original 1-*β* of 0.95 and indicating that the sample size of the study meets the sample size required for the mediation analysis. In addition, participants were recruited from 3 tertiary grade-A hospitals in China through questionnaires, and collected additional questionnaires to enhance the generalizability of the research findings, so as to minimize the deviation of the results caused by geography and hospital nature. In this way, a total of 518 questionnaires were distributed. After eliminating 12 questionnaires with common or obviously contradictory responses, we obtained 506 valid questionnaires. This resulted in an effective recovery rate of 97.7%.

### 3.3 Measurements

#### 3.3.1 The demographic characteristics questionnaire

The Demographic Characteristics Questionnaire was systematically developed and validated by the research team, comprising two distinct sections: demographic characteristics and clinical parameters. The demographic section encompassed eight key variables: age; gender; educational level; marital status; living status; occupation status; monthly income (RMB); and medical insurance payment method. The clinical domain incorporated five critical dimensions: medical insurance payment method; primary caregiver; time after enterstomy (months); whether needs to be reimbursed; are there any complications.

#### 3.3.2 The tolerance for mental pain scale (TMPS)

The Tolerance for Mental Pain Scale (TMPS) was initially proposed by Orbach et al. (2003) as a multidimensional assessment tool. Subsequent methodological refinement was conducted by Meerwijk et al. (2019), resulting in a streamlined 20-item version structured across two distinct conceptual dimensions: pain management and pain tolerance. The instrument employs a standardized 5-point Likert scale anchored by the following response options: 1 = “completely inconsistent,” 2 = “relatively inconsistent,” 3 = “neutral/uncertain,” 4 = “relatively consistent,” and 5 = “completely consistent.” Notably, items within the pain management subscale were reverse-scored to ensure psychometric validity. Total scores demonstrate a potential range from 10 to 50 points, with the original English version reporting excellent internal consistency (Cronbach’s *α* = 0.91). Cross-cultural adaptation was subsequently performed by Li et al. (2023) through rigorous translation and validation procedures. The Chinese version maintained satisfactory psychometric properties, achieving an overall Cronbach’s *α* coefficient of 0.832. Subscale reliability analysis revealed strong internal consistency for both dimensions: *α* = 0.894 for pain management and *α* = 0.856 for pain tolerance, confirming the instrument’s cross-cultural reliability.

#### 3.3.3 The ostomy adjustment inventory (OAI)

The Ostomy Adjustment Inventory (OAI) was initially developed by Simmons in 2009 (Simmons et al., 2009), grounded in the theoretical framework of the Self-Adaptation Scale for ostomy Patients. Subsequently, Gao and Yuan (2011) completed its cross-cultural adaptation and validation for Chinese populations in 2011, culminating in a refined instrument comprising 20 items organized into three distinct domains: persistent worry (9 items), acceptance (5 items), and positive life attitude (6 items). This tool employs a standardized 5-point Likert-type scoring system, where responses are quantified as follows: 0 = “strongly disagree,” 1 = “disagree,” 2 = “uncertain,” 3 = “agree,” and 4 = “strongly agree.” The total achievable score ranges from 0 to 80, with higher values reflecting enhanced psychosocial adaptation. Psychometric evaluations demonstrated robust reliability for the original OAI, yielding a Cronbach’s *α* coefficient of 0.93. Following cultural adaptation, the Chinese version retained strong internal consistency (Cronbach’s *α* = 0.886). Subscale reliability metrics further exhibited satisfactory performance across dimensions, with Cronbach’s *α* values ranging from 0.704 to 0.855, thereby confirming the instrument’s structural validity and measurement precision in cross-cultural applications.

#### 3.3.4 The stoma quality of life (S-QOL)

The Stoma Quality of Life (S-QOL) was originally developed by Prieto et al. (2005) in 2005 through rigorous psychometric validation, and has since undergone cross-cultural validation in multiple countries demonstrating robust psychometric properties. The questionnaire exhibits excellent internal consistency, with a Cronbach’s *α* coefficient of 0.92. This multidimensional assessment tool comprises 20 items distributed across four distinct domains: social interaction (6 items), physical/psychological wellbeing (3 items), interpersonal relationships (5 items), and stoma appliance management (6 items). Utilizing a standardized 4-point Likert-type scale where 1 = “always,” 2 = “sometimes,” 3 = “rarely,” and 4 = “never,” the instrument generates total scores ranging from 20 to 80 points. In 2011, Wu et al. (2011) conducted linguistic adaptation and psychometric evaluation of the Chinese version, yielding a Cronbach’s *α* coefficient of 0.893, confirming its measurement reliability in Chinese populations. A higher total score reflects superior QOL outcomes among enterstomy patients.

#### 3.3.5 The stoma self-care scale (SSCS)

The Stoma Self-care Scale-Early Edition (SSCS-EE) was developed through systematic integration of two validated instruments: the foundational Stoma Self-care Scale proposed by Piwonka and Merino (1999) and the operationalized enterstomy Care Measurable Skills framework established by Johnson and Porrett (2005). This preliminary version was subsequently adapted by Zhang, J. E. et al. (2010) through rigorous cross-cultural validation processes tailored to the unique clinical characteristics of permanent colostomy patients in China, ultimately yielding a final 10-item instrument. The assessment employs a standardized 5-point Likert scale, with response anchors defined as follows: 1 = “very unskilled,” 2 = “unskilled,” 3 = “moderately skilled,” 4 = “proficient,” and 5 = “highly proficient.” Following psychometric evaluation, the scale demonstrated strong internal consistency (Cronbach’s alpha = 0.988), with higher total scores indicating greater self-care competency - higher total scores indicating superior self-care mastery.

### 3.4 Data collection

Participants were recruited from 3 tertiary grade-A hospitals in China. The investigation was conducted with the prior approval of the hospital administrators. First, the chief nurse assisted in conducting the inquiry with the prior consent of the hospital management, using the department as the unit. Second, before the investigation, the researchers informed the participants about the study’s aim, importance, and privacy. The study participants were free to decline or leave at any time, and the information collected will only be used for academic research and not for profit. In this manner, the informed consent form was signed before the research, and the participant’s consent was acquired. In-person, the surveys were completed in around 15–20 min, and participants were asked to fill in the questionnaires face-to-face, after which they were immediately collected and verified. In this study, a total of 518 questionnaires were collected within the specified time, and after excluding 12 invalid questionnaires, a total of 506 valid questionnaires were obtained, with an effective recovery rate of 97.7%.

### 3.5 Statistical analysis

Two researchers recorded and analyzed the raw data using Epidata 3.1 and IBM SPSS 26.0. Descriptive statistics (numbers and percentage distributions) were used to describe the demographic characteristics. Measurement data following a normal distribution were described using mean ± standard deviation (M ± SD). Group comparisons were performed using two independent sample *t*-tests or one-way ANOVA for normally distributed data. For non-normally distributed measurement data, the median and interquartile ranges were used, and group comparisons were conducted using Mann–Whitney U or Kruskal–Wallis tests. The Pearson methodology was utilized to analyze the correlation between psychological pain tolerance, psychosocial adaptation, quality of life, and self-care, with statistical significance set at *p* < 0.05 (two-tailed). In this study, the assumptions for the correlation tests were met, and the variables were normally distributed and the two variables in each analysis were linearly associated. Prior to conducting multiple linear regression, key assumptions were tested. Multicollinearity was assessed using variance inflation factor (VIF), with values below 5 indicating no serious multicollinearity. The normality of residuals was examined using P–P plots, and homoscedasticity was checked via scatterplots of standardized residuals against predicted values. All assumptions were met, supporting the validity of the regression results.

### 3.6 Ethics considerations

The study protocol was approved by the Medical Ethics Committee of Hengyang Central Hospital (HYZXYY-2023-05-027). Written informed consent was obtained from each participant. All procedures were performed per the principles of the Declaration of Helsinki and relevant guidelines and regulations in China. After obtaining permission from hospital administrators, the researchers approached the participants with the help of the head nurses. The participants were given the right to refuse or withdraw from this study. The questionnaires were designed to ensure anonymity and confidentiality, with no identifying marks, names, or numbers linked to the participants. The data obtained were only used for academic research and not commercial purposes.

## 4 Results

### 4.1 Demographic characteristics among hospitalized patients with enterstomy

A cross-sectional investigation was conducted, prospectively enrolling 506 consecutive enterstomy patients from three tertiary grade-A hospitals in China. The study population demonstrated a pronounced gender disparity, with male participants representing 22.13% (*n* = 112) of the cohort compared to 77.87% (*n* = 394) female representation. Comprehensive demographic profiling, including baseline clinical characteristics and socioeconomic parameters, was systematically summarized through standardized data collection protocols, as detailed in Table 1.

**Table 1:** The demographic characteristics and univariate analysis of psychological pain tolerance among hospitalized patients with enterstomy (*n* = 506, M ± SD).

Characteristics		*n*	%	Total score of TMPS	*t*/*F*	*p*
Age (years)	<40	452	89.33	33.22 ± 10.52	3.478	0.033
	40–60	30	5.93	31.00 ± 7.42		
	>60	24	4.74	28.58 ± 11.94		
Gender	Male	112	22.13	34.48 ± 10.58	3.412	<0.001
	Female	394	77.87	30.81 ± 10.36		
Education level	Primary and below	11	2.17	30.00 ± 13.96	2.054	0.105
	Junior high school	9	1.78	24.44 ± 10.92		
	Technical secondary school/high school	15	2.96	30.47 ± 6.06		
	College or above	471	93.08	32.43 ± 10.39		
Marital status	Unmarried	47	9.29	31.36 ± 8.15	0.159	0.853
	Married	442	87.35	32.26 ± 10.64		
	Divorced/widowed	17	3.36	32.29 ± 10.65		
Living status	Living with other people	380	75.10	30.78 ± 10.16	−2.261	0.024
	Living alone	126	24.90	33.89 ± 11.14		
Monthly income (RMB)	<2,000	317	62.65	34.75 ± 10.47	5.897	<0.001
	2,000–3,999	57	11.26	32.28 ± 11.21		
	4,000–6,000	35	6.92	29.21 ± 8.62		
	>6,000	97	19.17	27.31 ± 9.89		
Occupation status	Enterprise/institution staff	125	24.70	32.38 ± 10.74	0.197	0.822
	Worker	35	6.92	31.14 ± 8.25		
	Unemployed	346	68.38	32.21 ± 10.52		
Medical insurance payment method	Medical insurance	399	78.85	32.20 ± 10.29	0.025	0.975
	New rural cooperative	51	10.08	31.88 ± 11.73		
	Self-paying	56	11.07	32.30 ± 10.31		
Primary caregiver	Mate	56	11.07	30.13 ± 9.22	1.069	0.362
	Offspring	27	5.34	31.63 ± 10.66		
	Parents	289	57.11	32.75 ± 10.22		
	Others	134	26.48	31.92 ± 11.23		
Time after enterstomy (months)	1–3	249	49.21	28.92 ± 10.73	4.886	<0.001
	4–6	123	24.31	30.04 ± 9.62		
	7–12	72	14.23	31.19 ± 9.95		
	>12	62	12.25	34.61 ± 11.18		
Whether needs to be reimbursed	Yes	241	47.63	30.38 ± 9.67	−2.361	0.019
	No	265	52.37	33.91 ± 11.03		
Are there any complications	No	377	74.51	33.12 ± 10.49	0.812	0.433
	Peristomy dermatitis	73	14.43	29.30 ± 9.49		
	Parastomal hernia	14	2.77	32.50 ± 11.35		
	Stricture of stoma	8	1.58	31.25 ± 7.80		
	Bleeding from stoma	18	3.56	30.50 ± 10.44		
	Others	16	3.16	25.13 ± 9.02		

### 4.2 Scores of psychological pain tolerance, psychological adaptation, quality of life, and self-care among hospitalized patients with enterstomy

The total score of psychological pain tolerance was 32.18 ± 10.42, with a mean score of 3.22 ± 1.04. Among the dimensions of TMPS, “pain tolerance” had the highest average score (3.35 ± 1.23), while “pain management” had the lowest average score (3.08 ± 1.27).

The total score of psychological adaptation was 44.77 ± 18.56, with an overall mean of 2.24 ± 1.03. Among the three dimensions of the OAI, “Acceptance” had the highest average score (2.45 ± 1.03), while “Persistent worry” had the lowest (2.04 ± 0.92). The average scores for the “Positive life attitude” dimensions was 2.36 ± 1.10.

The total score of quality of life was 49.18 ± 17.90, and the average score was 2.46 ± 0.90. Among the four dimensions of the S-QOL, “Physical/psychological wellbeing” had the highest average score (2.50 ± 0.94), while “Stoma appliance management” had the lowest (2.43 ± 0.96). The average scores for the other dimensions were as follows: “Social interaction” (2.47 ± 0.90), and “Interpersonal relationships” (2.46 ± 0.93).

The total score of self-care was 33.58 ± 11.42, and the average score of SSCS was 3.36 ± 1.14.

The scores of TMPS, OAI, S-QOL, and SSCS were shown in Table 2.

**Table 2:** The scores of psychological pain tolerance, psychological adaptation, quality of life, and self-care among hospitalized patients with enterostomy (*n* = 506, M ± SD).

Dimensions	Number of items	Number of items	Average score of items	Ranking
TMPS tolerance total score	20	32.18 ± 10.42	3.22 ± 1.04	–
Pain management	10	15.41 ± 6.33	3.08 ± 1.27	2
Pain tolerance	10	16.77 ± 6.16	3.35 ± 1.23	1
OAI total score	20	44.77 ± 18.56	2.24 ± 1.03	–
Persistent worry	9	18.39 ± 5.68	2.04 ± 0.92	3
Acceptance	5	12.24 ± 5.63	2.45 ± 1.03	1
Positive life attitude	6	14.14 ± 6.79	2.36 ± 1.10	2
S-QOL total score	20	49.18 ± 17.90	2.46 ± 0.90	–
Social interaction	6	14.81 ± 5.41	2.47 ± 0.90	2
Physical/psychological wellbeing	3	7.50 ± 2.83	2.50 ± 0.94	1
Interpersonal relationships	5	12.29 ± 4.63	2.46 ± 0.93	3
Stoma appliance management	6	14.58 ± 5.74	2.43 ± 0.96	4
SSCS total score	10	33.58 ± 11.42	3.36 ± 1.14	–

### 4.3 Univariate analysis of psychological pain tolerance among hospitalized patients with enterstomy

The result of univariate analysis showed that age, gender, living status, monthly income, time after stomy, and whether needs to be reimbursed emerged as statistically significant factors associated with psychological pain tolerance among hospitalized patients with enterstomy (all *p* < 0.05), as shown in Table 1.

### 4.4 Correlations among psychological pain tolerance, psychological adaptation, quality of life, and self-care among hospitalized patients with enterstomy

The total psychological pain tolerance score was positively correlated with the total scores for psychological adaptation (*r* = 0.674, *p* < 0.01), quality of life (*r* = 0.542, *p* < 0.01), and self-care (*r* = 0.513, *p* < 0.01). Significant positive correlations were also observed across all subscales of these measures, with correlation coefficients ranging from 0.346 to 0.644 (all *p* < 0.01), as shown in Table 3.

**Table 3:** The correlations among psychological pain tolerance, psychological adaptation, quality of life, and self-care among hospitalized patients with enterstomy (*n* = 506, *r*).

Item	TMPS total score	Pain management	Pain tolerance
OAI total score	0.674**	0.573**	0.554**
Persistent worry	0.638**	0.644**	0.421**
Acceptance	0.552**	0.386**	0.540**
Positive life attitude	0.545**	0.370**	0.545**
S-QOL total score	0.542**	0.515**	0.390**
Social interaction	0.546**	0.501**	0.413**
Physical/psychological wellbeing	0.519**	0.489**	0.378**
Interpersonal relationships	0.514**	0.493**	0.366**
Stoma appliance management	0.504**	0.496**	0.346**
SSCS total score	0.513**	0.399**	0.458**

**p < 0.01.

### 4.5 Multiple linear regression analysis of psychological pain tolerance among hospitalized patients with enterstomy

In this study, the psychological pain tolerance composite score of hospitalized enterstomy patients served as the dependent variable. Independent variables comprised six statistically significant sociodemographic and clinical characteristics identified through univariate analysis (age, gender, living status, monthly income, time after enterstomy, and whether needs to be reimbursed), along with variables demonstrating statistical significance in correlation analysis. Variable coding specifications are detailed in Table 4, with continuous variables retained in their original continuous form. A multiple linear regression model was constructed to identify significant correlates of psychological pain tolerance among enterstomy patients, ultimately incorporating nine variables into the final equation.

**Table 4:** Independent variable assignment (*n* = 506).

Project	Assignment method
Age	<40 = 1, 40–60 = 2, >60 = 3
Gender	Male = 0, Female = 1
Living status	Living with other people = 0, live alone = 1
Monthly income	<2,000 = 1, 2,000–3,999 = 2, 4,000–6,000 = 3, >6,000 = 4
Time after stomy	1–3 = 1, 4–6 = 2, 7–12 = 3, >12 = 4
Whether needs to be reimbursed	Yes = 0, No = 1
Are there any complications	Take “none” as dummy variable, No = (0, 0, 0, 0, 0, 0), Peristomy dermatitis = (0, 1, 0, 0, 0, 0), Parastomal hernia = (0, 0, 1, 0, 0, 0), Stricture of stoma = (0, 0, 0, 1, 0), Bleeding from stoma = (0, 0, 0, 0, 1, 0), Others = (0, 0, 0, 0, 0, 1)
OAI total score	Original entry
S-QOL total score	Original entry
SSCS total score	Original entry

The results of multiple linear regression analysis revealed seven key variables significantly associated with psychological pain tolerance (*p* < 0.05): age, gender, living conditions, time after enterstomy, psychological adaptation, quality of life, and self-care, indicating that these were the main factors associated with psychological pain tolerance among hospitalized patients with enterstomy, explaining 45.8% of the total variation, as shown in Table 5.

**Table 5:** Multiple linear regression analysis of psychological pain tolerance among hospitalized patients with enterostomy (*n* = 506).

Project	*B*	*SE*	*β*	*t*	*p*
Constant quantity	64.878	7.325	–	8.857	<0.001**
Age	−7.486	1.632	−0.345	−4.587	<0.001**
Gender	−6.732	1.458	−0.345	−4.617	<0.001**
Living status	4.779	1.310	0.298	3.649	<0.001**
Monthly income	0.893	1.213	0.024	0.736	0.385
Time after stomy	3.421	0.962	0.276	3.556	<0.001**
Whether needs to be reimbursed	1.143	1.794	0.015	0.637	0.617
Are there any complications	0.838	0.112	0.585	7.455	<0.001**
OAI total score	0.457	0.108	0.317	4.232	<0.001**
S-QOL total score	0.725	0.102	0.486	7.108	<0.001**
SSCS total score	0.522	0.137	0.298	5.292	<0.001**

**p < 0.01, *R* = 0.686, *R*^2^ = 0.471, adjusted *R*^2^ = 0.458, *F* = 117.251, *p* < 0.001.

## 5 Discussion

### 5.1 Status quo of psychological pain tolerance among hospitalized patients with enterstomy

Hospitalized patients with enterostomy in this study had a mean psychological pain tolerance score of 3.22 ± 1.04. When compared with the scale’s median value of 3, this indicates moderate-level psychological pain tolerance among the study population, potentially associated with heightened disease threat perception following enterostomy surgery. The psychological pain assessment comprised two distinct dimensions: active pain management and passive pain tolerance, corresponding to different coping mechanisms. Pain management was operationally defined as implementing strategies to eliminate or mitigate distress, whereas pain tolerance represented passive endurance with anticipated spontaneous resolution (Meerwijk et al., 2019). Notably, the mean score for pain management items (3.08 ± 1.27) demonstrated comparatively lower performance relative to the overall scale mean, while pain tolerance items (3.35 ± 1.23) exceeded the overall mean. This divergence suggests that when psychological distress remains unmanaged through active strategies, patients’ capacity for passive tolerance diminishes substantially, potentially elevating risks for depressive symptoms and suicidal ideation (Landi et al., 2020).

These findings underscore the clinical imperative for nursing professionals to systematically evaluate the decision-making equilibrium in psychological pain tolerance among hospitalized patients with enterostomy. Such assessment facilitates identification of implementation barriers in pain management protocols and enables the development of dimension-specific interventions. By addressing both active coping mechanisms and passive endurance capacities through targeted strategies, healthcare providers can effectively optimize psychological pain regulation and reduce overall distress levels in this vulnerable population.

### 5.2 Factors associated with psychological pain tolerance in hospitalized patients with enterostomy

#### 5.2.1 Demographic characteristics

Older hospitalized patients with enterostomy exhibited significantly lower psychological pain tolerance levels. This phenomenon may be associated with the clinical characteristics of older enterostomy patients, who typically experienced a higher prevalence of chronic comorbidities and persistent disease-related pain, coupled with diminished life expectations and reduced social responsibilities. These cumulative factors collectively contributed to decreased pain tolerance thresholds, a finding corroborated by Melum et al.’s (2023) prospective cohort study of 21,837 hospitalized patients (including 311 stroke survivors). Their results revealed that stroke patients exhibited significantly reduced physiological and psychological pain tolerance compared to non-stroke counterparts. Furthermore, Lutzman and Sommerfeld’s (2024) identified psychological distress as a complication of chronic maladaptive avoidance coping strategies, particularly associated with elevated suicide risks in elderly males. In contrast, younger enterostomy patients and male populations demonstrated greater health awareness regarding disease management, exhibited enhanced decision-making capacities in health behavior regulation, and displayed stronger adherence to proactive pain management protocols. Based on these findings, we recommend that clinical nursing teams prioritize targeted psychological interventions for elderly and female enterostomy patients. Specifically, evidence-based cognitive behavioral approaches should be implemented to modify maladaptive cognitive patterns and enhance psychological pain tolerance through structured coping strategy training.

Furthermore, our analysis revealed that solitary-living enterostomy patients exhibited significantly higher psychological pain tolerance compared to those cohabiting with family members. Paradoxically, the familial caregiving dynamics experienced by cohabiting patients appeared to exacerbate their perceived burden while diminishing autonomous health management capacities-findings which contrasts with the findings reported by Xu (2022). A positive correlation emerged between stoma duration and psychological resilience, with prolonged stoma duration corresponding to enhanced pain tolerance. This temporal pattern may be attributed to initial deficiencies in disease-specific knowledge and overly optimistic prognostic expectations during early postoperative stages. As stoma duration progresses, patients typically undergo psychological adaptation to altered self-image while developing practical stoma management competencies. These acquired skills effectively reduce stoma-related complications and enhance self-efficacy, collectively contributing to improved psychological resilience. This temporal adaptation pattern aligns with Pennings et al.’s longitudinal observations (Pennings et al., 2021), which documented patients’ progressive acquisition of stoma-related knowledge and successful lifestyle adaptation, ultimately yielding improved quality-of-life metrics and pain tolerance thresholds. These findings underscore the clinical imperative for targeted interventions focusing on two vulnerable subgroups: early postoperative patients (particularly those within familial caregiving contexts) and cohabiting individuals. Implementation strategies should emphasize family-centered psychological support coupled with structured stoma education programs to optimize psychological adaptation trajectories.

#### 5.2.2 Psychosocial adaptation is positively associated with psychological pain tolerance

Hospitalized patients with enterostomy in this study had a mean psychological adaptation score of 44.77 ± 18.56, aligning with previous observations reported by Tai et al. (2023). Notably, psychosocial adaptation scores revealed a significant positive correlation with psychological pain tolerance levels in this population. Multiple linear regression analysis confirmed psychological adaptation as a critical variable positively associated with psychological pain tolerance. Importantly, optimal adaptation encompasses not merely physical adjustment but also psychosocial integration, as postoperative enterostomy precipitates fundamental alterations in patients’ lifestyle patterns, self-perception, and interpersonal dynamics (Zuo, 2024). These profound changes frequently engender persistent anxiety, depressive symptoms, and maladaptive coping mechanisms, ultimately compromising adaptation outcomes.

To enhance clinical outcomes, we propose a stratified intervention protocol: First, implement phased adaptation objectives with evidence-based behavioral targets. Second, employ case-based learning through exposure to successful adaptation models to facilitate cognitive restructuring. Third, utilize motivational interviewing techniques with concrete exemplars to reinforce self-efficacy. Concurrently, healthcare providers should conduct systematic psychological evaluations during hospitalization using validated assessment tools. This enables timely identification of psychological distress manifestations and subsequent implementation of targeted cognitive-behavioral interventions. Such comprehensive approaches may effectively accelerate the achievement of optimal psychosocial adaptation in enterostomy patients.

#### 5.2.3 Quality of life is positively associated with psychological pain tolerance

The findings of this study demonstrated a significant positive correlation between quality of life and psychological pain tolerance among hospitalized patients with enterstomy, indicating that higher quality of life is associated with greater psychological pain tolerance. Specifically, the quality of life assessment for these patients encompassed four critical domains: social interaction capacity, physical-mental composite status, kinship relationship quality, and enterstomy pouch management competence. This multidimensional framework aligns with previous investigations: Xiao and Liu (2023) established through their family-centered nursing model research that optimized social engagement and strengthened familial bonds effectively mitigate psychological distress intensity, with superior social support systems demonstrating protective effects against social alienation induced by psychological pain. Complementarily, Zhao and Liu (2023) validated that evidence-based enterstomy pouch management protocols not only ameliorate stigma-related negative affect and psychological discomfort but also reduce stoma-related dermatological complications, thereby collectively enhancing psychological pain tolerance. Notably, the quality of life measurement in this cohort essentially reflects patients’ perceived social support adequacy and self-management competency in enterstomy care. Higher quality of life scores were associated with more effective psychological pain prevention and control mechanisms. Implementation of targeted health interventions - including family-mediated support systems, peer-assisted counseling, and evidence-based stoma care education-could substantially improve health behavior decision-making capacities in this population. Clinical implications emphasize the necessity for comprehensive assessment of patients’ enterstomy management proficiency and their tripartite support needs (social-familial-individual). A multidimensional intervention strategy integrating these elements is recommended to optimize life quality metrics and concomitantly alleviate psychological distress among hospitalized patients with enterstomy.

#### 5.2.4 Self-care is positively correlated with psychological pain tolerance

A significant positive correlation was found between self-care and psychological pain tolerance. Self-care, defined as an individual’s capacity for autonomous decision-making regarding health management, fundamentally influences enterostomy patients’ disease comprehension, self-management efficacy, and psychological adaptation (Villa et al., 2019). Enhanced self-care competence has been consistently associated with improved quality of life and mental health outcomes in this population (Zhou et al., 2024). Specifically, our data demonstrated that participants scoring higher on enterostomy self-care assessments exhibited enhanced psychological pain tolerance. This observation aligns with Collado-Boira et al.’s (2021) multi-center study of 139 enterostomy patients, which established significant associations between self-care ability, health-related quality of life improvements, and reduced psychological distress. These findings collectively suggest that systematic implementation of evidence-based self-care strategies may facilitate pain mitigation and disease adaptation. Supporting this perspective, Ko et al. (2023) implemented a multimedia educational intervention that significantly enhanced both quality of life and therapeutic confidence through self-care optimization. Consequently, clinical practitioners should prioritize comprehensive assessment of self-care capabilities among hospitalized patients with enterstomy, employing information technology to develop personalized, multidimensional self-care protocols that address specific patient needs while maintaining therapeutic rigor.

#### 5.2.5 Clinical implications

The findings of this study have practical implications for nursing practice. For instance, nurses could use the Tolerance for Mental Pain Scale (TMPS) during hospitalization to identify patients with low psychological pain tolerance. Those scoring low could be offered targeted interventions such as cognitive-behavioral therapy (CBT) sessions to enhance their emotional regulation and coping skills. Additionally, patients with poor self-care abilities might benefit from structured education programs focused on stoma management, which could indirectly improve their psychological pain tolerance by boosting self-efficacy and reducing daily stressors. These examples illustrate how integrating psychological pain tolerance assessment into routine care can guide personalized nursing interventions, ultimately improving patient outcomes.

### 5.3 Limitations

There are several limitations to this study. First, a convenience sampling method was employed, enrolling only 506 hospitalized patients with enterstomy from three tertiary grade-A hospitals in China. This sampling approach may result in an unrepresentative sample and potentially biased, non-generalizable findings. Future research should address this limitation by using random, multi-center sampling with a larger sample size. Second, the study’s cross-sectional design limits the ability to establish causal relationships. Longitudinal research is needed to explore further the mechanisms underlying the relationships identified in this study. Third, the sample had a pronounced female predominance (77.87%), which may limit the generalizability of the findings to male patients with enterstomy. Future studies should aim for a more balanced gender representation to enhance the external validity of the results. Future studies should include a larger and more diverse sample among hospitalized patients with enterstomy and investigate the factors influencing psychological pain tolerance in more depth.

## 6 Conclusion

The psychological pain tolerance among hospitalized patients with enterstomy was moderate, which need to be further improved. Age, gender, living conditions, time after enterstomy, psychological adaptation, quality of life, and self-care were the main associated factors of psychological pain tolerance among hospitalized patients with enterostomy. It is suggested that nursing staff should formulate personalized intervention programs according to the associated factors of psychological pain tolerance among hospitalized patients with enterstomy, so as to improve the quality of life among hospitalized patients with enterstomy.

## Data Availability

The original contributions presented in the study are included in the article/supplementary material, further inquiries can be directed to the corresponding authors.
